# Barriers to healthcare access during pregnancy and after birth for adolescent girls living with disability in Sierra Leone: a qualitative study

**DOI:** 10.1136/bmjopen-2025-109084

**Published:** 2026-06-02

**Authors:** Philemon Kamara, Mangenda Kamara, Lucy November, Jane Sandall, Prince T Williams, Haja Wurie, Cristina Fernandez Turienzo

**Affiliations:** 1 Lifeline Nehemiah Projects, Freetown, Sierra Leone; 2 University of Sierra Leone, Freetown, Western Area, Sierra Leone; 3 Department of Women & Children’s Health,School of Life Course & Population Health Sciences, Faculty of Life Sciences & Medicine, King’s College London, London, UK

**Keywords:** PUBLIC HEALTH, Adolescents, Pregnancy, Disabled Persons

## Abstract

**Abstract:**

**Background and objective:**

In 2017, Lifeline Nehemiah Projects in Sierra Leone launched 2YoungLives, a mentoring initiative for vulnerable pregnant adolescents, including those living with disabilities. Drawing from the social model of disability, we aimed to investigate the exacerbated disabling barriers which prevent these girls and their babies from accessing available healthcare and develop comprehensive solutions to improve their access.

**Design:**

Qualitative methods were used in this study.

**Setting:**

Participants were invited to participate in face-to-face interviews in one district in Sierra Leone.

**Participants:**

Six pregnant or postnatal adolescents living with disability, alongside four caregivers and five stakeholders from various organisations participated in semi-structured interviews employing thematic analysis.

**Results:**

We identified four key themes: (1) discrimination and financial barriers within the healthcare system, despite laws ensuring free healthcare for persons living with disabilities, (2) societal stigma manifested through abandonment by families and inadequate support, (3) lack of understanding of disability issues, particularly of those with intellectual impairments, leading to stigmatisation and exclusion, and (4) infrastructural limitations which hinder accessibility to essential services, with many facilities remaining non-compliant with disability regulations.

**Conclusion:**

Adolescent girls living with disability during pregnancy and after birth in Sierra Leone face barriers to accessing healthcare, including caregiver ignorance, lack of autonomy, disabling services, social stigma and ineffective policy implementation, despite existing supportive laws. These findings keep those women who are arguably the most vulnerable, adolescent and living with a disability, from accessing perinatal healthcare, exacerbating their risk and that of their babies. Solutions include the need to enforce disability-inclusive policies and infrastructure adaptations, awareness and training for healthcare providers and community advocacy to break down social stigma.

**STRENGTHS AND LIMITATIONS OF THIS STUDY**

- This study conducted semistructured interviews with 15 participants in Sierra Leone to comprehensively explore their experiences.
- The inclusion of caregivers and stakeholders provides triangulated perspectives on the barriers to care.
- The application of the social model of disability to the thematic analysis ensured an structural focus on the data, rather than a medicalised interpretation.
- The study is limited by its small sample size and single-district setting, which may limit transferability to other contexts.
- While this was a small sample of girls with disabilities, they represented the eligible girls within the study sites, providing qualitative depth and contextual richness.

## Background

 Sierra Leone has made commendable progress in reducing maternal mortality, with the maternal mortality ratio declining from 1682 deaths per 100 000 live births in 2000 to 354 deaths per 100 000 live births recorded in 2023.^1^ Despite this significant improvement, the rate remains among the highest globally and continues to pose a major public health challenge. The Government of Sierra Leone remains committed to accelerating progress, with an ambitious target of fewer than 300 maternal deaths per 100 000 live births by 2025.^2^ Adolescent girls account for about 20–40% of all maternal deaths; over one-third of women aged 20–24 gave birth before age 18 and 10% gave birth before age 16.^3 4^ Infants born to adolescent mothers also face heightened risks of illness and death.^3^ In 2017, Lifeline Nehemiah Projects, a non-governmental organisation (NGO) based in Sierra Leone, developed and piloted 2YoungLives, a mentoring scheme for vulnerable pregnant adolescent girls up to 1 year after birth. This led to a pilot cluster-randomised controlled trial of 2YoungLives^5^ which showed a 48% reduction in a composite of maternal and perinatal death in the intervention^6^ and evidence of how 2YoungLives impacted on girls’ economic, social and educational empowerment. While the trial demonstrated significant success, our field observations revealed that adolescents with disabilities remained a highly vulnerable subgroup facing multiple struggles and gaps in care.

Disability prevalence for Sierra Leone is 4.3% in the general population, with a lack of disaggregated data for the adolescent age group.^7^ People living with disabilities are at higher risk of living in poverty, being unemployed and having less access to healthcare compared with the general population.^8^ If poverty is a major cause for exclusion from healthcare services, then having an impairment compounds this exclusion. As part of our work, the 2YoungLives team has encountered several barriers fundamental to accessing healthcare services faced by pregnant adolescents with impairments; for example stigmatised behaviour towards girls with learning disabilities, both within communities and health services, a lack of recognition of communication difficulties and a lack of knowledge about transmission of disabilities from mother to baby, leading to incorrect advice about breastfeeding. This prompted our decision to conduct this qualitative study to further explore these barriers and to proffer holistic solutions which may help to address them.

This study is informed by the social model of disability,^9^ which views disability as arising not just from individual impairments but from socially constructed barriers, including stigma, exclusionary attitudes and inaccessible environments. In contrast to medical or individual, the social model draws attention to how health systems, social norms and policy environments produce disability-related disadvantage. This framework is particularly relevant for pregnant adolescents with disabilities in Sierra Leone, whose experiences are shaped by intersecting barriers related to age, gender, poverty and social marginalisation. Applying the social model allows for an analysis that considers structural and institutional factors influencing access to sexual and reproductive health services, rather than framing challenges as individual limitations. The study thus sets out to answer key questions about the attitudinal, institutional, communication and environmental barriers which adolescent pregnant girls with disabilities face when accessing healthcare despite the Free Healthcare Initiative (FHCI) and the National Commission for People with Disabilities (NCPD) which was established to ensure the well-being of people with disabilities through the enactment of the Person with Disability Act 2011.^10^

This study was part of the seed-funded capacity building activities of a global health research group funded by the UK’s National Institute of Health and Care Research, focused on innovations to reduce maternal and perinatal mortality and build research capacity in Sierra Leone.^11^

## Methods

This study used a qualitative design, using semistructured interviews to allow for an in-depth understanding of barriers to healthcare during pregnancy and after birth for adolescent girls living with disability, and to demystify the taboos and misunderstandings that enshroud these groups in Sierra Leone. These questions were: *What limits adolescent girls with disabilities from accessing healthcare? What can be done to improve access to healthcare for pregnant girls with disabilities? What is the awareness level for disability issues in the general public and among health professionals? Are the policies (FHCI and Disability Act) having their desired impact?*

The study was conducted in a district town in Sierra Leone with a tertiary hospital. Due to the sensitivity of the data, the sites are not named. We secured a private room at the hospital as a setting for interviews, and gave participants the choice of this setting or a setting of their choice. We report this study based on the Consolidated criteria for Reporting Qualitative Research (COREQ).^12^

### Participants and recruitment

Participants were included if they: (1) were girls under 18 years of age, with impairments who were pregnant or in the postpartum year, (2) were family, friends or guardians of these girls, (3) were stakeholders representing services and organisations accessed by people living with disabilities. Girls and family members or guardians were identified by the researchers’ informal network in the district headquarter town selected for the research. Stakeholders were identified through networks of relevant institutions, including health services, local councils, faith-based organisations and disability advocacy bodies. Written informed consent was obtained for all participants invited for interviews. For participants with learning disabilities and those under 16, written consent was sought from their parent or guardian, using a personal approach by the research assistant (2YoungLives founder, gender specialist, PhD student) to explain the research and pretested information sheets in Krio or other local language, with opportunity for questions. Assent was sought from the under 16 years old.

Both convenience sampling and purposive sampling techniques were used: interviewing anyone who met the inclusion criteria and wanted to participate in the study after reading the participant information sheet (convenience sampling); and through intense collaboration with community leaders and organisations to recruit as diverse a sample of participants from different cultural, socio-economic and religious backgrounds as possible (purposive sampling). Six girls, four caregivers and five stakeholders participated in the study. Four out of the six girls had a learning disability (one of whom also had a spinal deformity, and one of whom was non-verbal), the two girls with physical disabilities both had limb deformities from contracting polio as children. Although the sample of six girls with disabilities is small, it captured all eligible participants within the study site and, when triangulated with caregiver and stakeholder perspectives, provided sufficient thematic depth and data saturation for this exploratory qualitative study.

### Data collection

Interviews took place at their preferred location; four in their homes and two in the private room at the district hospital following antenatal clinic appointments. Some participants requested to be interviewed without the presence of caregivers; these preferences were fully respected, and girls were given the option to choose the setting and whether a trusted non-participant observer would be present. In addition, four caregivers and five stakeholders were interviewed. Stakeholders represented a range of perspectives and institutions; the district council (DC), a senior healthcare worker (matron), the inter-religious council (RC), the NCPD and an NGO supporting persons with disabilities.

All participants received information sheets in advance or immediately before interviews. For adolescent girls and caregivers, information was provided in English and orally translated into their preferred local language (Krio or Mende). Consent (and assent for under 16) procedures emphasised that participation was voluntary and confidential, with no consequences for choosing not to participate. This assurance was especially crucial for the adolescent girls, who expressed concerns about legal repercussions or prior unsuccessful engagements with law enforcement. Participants were informed that they could withdraw from the study at any point within agreed time frames, and data confidentiality was guaranteed. To further protect privacy, we have withheld district-level identifiers in reporting.

All interviews were conducted by a trained local female researcher (MK), a gender specialist with experience in qualitative methods. Her role helped foster trust and allowed participants to speak freely about sensitive gender and disability issues, particularly topics culturally inappropriate to discuss with male interviewers. Interviews were conducted in Krio or Mende according to participant preference. Each participant received a reimbursement of 50 leones to cover time and travel expenses.

Semistructured interviews lasted between 15 and 25 min and were conducted face to face between April 2023 and November 2023. Interviews were audio-recorded, translated and transcribed and de-identified by an independent transcriber fluent in both Krio and Mende. Field notes were taken during the interviews (ie, observations of the environment, attitudes of participants, neighbours or friends). Transcriptions were checked for accuracy by another researcher (LN). The interview schedule, which was co-designed with girls living with disabilities, family members and caregivers, aimed to explore girls’ experiences of accessing maternity care during pregnancy and the postnatal period. The interview schedule was used flexibly, allowing participants to share their stories in the order they preferred; it served as a guide, prompting further discussion only when necessary.

### Data analysis

Interview data was analysed by two researchers (MK, PK) following Braun and Clarke’s six stages to thematic analysis: familiarising with data, generating initial codes, searching for themes, reviewing themes, defining themes and writing up.^13^ Data analysis was conducted concurrently with data collection to develop and relate themes and categories, thereby deepening the understanding of the phenomenon by adding meaning to the data. To increase interpretive rigour and validity, two transcripts were coded by one of the researchers’ supervisors (LN). There was strong agreement among the authors regarding the coding process. Analysis was guided by the social model of disability, focusing on how social attitudes, health system practices and environmental constraints shaped experiences of pregnancy and healthcare access. This approach enabled attention to structural barriers and exclusionary practices rather than individual functional limitations. The analysis followed an inductive approach, organising data into themes. Each theme was reviewed, with its relevance to the entire dataset considered. The analytical-inductive process was further enhanced by the field researchers’ reflexivity, team discussions and the use of a field diary.

### Community engagement and involvement

Community engagement and involvement (CEI) has been central to the development of 2YoungLives generally, and to this study specifically. A model of CEI developed by Lifeline Nehemiah Projects uses an iterative process of respectful dialogue with a range of local leaders and stakeholders, alongside co-production with local girls, women and community members, allowing new learning to be continuously embedded into the intervention.^14^ A similar model was used in this substudy, where the additional barriers to healthcare settings by girls with disabilities was highlighted by community women acting as mentors for the 2YoungLives project. Discussions with these women developed the research questions and helped formulate the most appropriate ways of engaging girls with disabilities and their families.

## Results

The characteristics of all participants are presented in table 1. The six girls are referred to with pseudonyms; Adama, Balu, Clara, Dawn, Effe, Fatmata; and the caregivers are identified in relation to the girl they care for. Stakeholders are identified as SHs, followed by their role.

**Table 1:** Characteristics of participants

Girls and caregivers		
Pseudonym	Disability	Caregiver
Adama	Learning disability	Adama’s caregiver
Balu	Learning disability	Balu’s caregiver
Clara	Physical disability	
Dawn	Learning disability, non-verbal	Dawn’s caregiver
Effe	Physical disability	
Fatmata	Learning disability and spinal deformity	Fatmata’s caregiver
SHs		
SH-DC	Representative of the district council	
SH-HCW	Senior healthcare worker	
SH-RC	Representative of inter-religious council representative	
SH-NCPD	Representative of National Commission for People with Disabilities	
SH-NGO	Representative of non-governmental organisation supporting persons with disabilities	

SH, stakeholder.

Four key themes regarding barriers to care were identified: lack of understanding of disability issues; social stigmatisation of people living with disabilities; infrastructural limitations; and policies and protocols. Table 2 summarises these four themes, specifying which participants contributed to each, before they are explored in detail below.

**Table 2:** Theme summary in relation to social model of disability and participants

Theme	Theme’s relation to social model of disability/brief description	Participants who contributed to this theme
Policy/practice disconnect	Institutionalised exclusion. Despite policies to protect the rights of people with disabilities, they are routinely charged for what should be free healthcare.	Adama’s caregiver, Balu’s caregiver, Fatmata, SH-HWC, SH-DC
Social stigmatisation for girls living with disabilities	Social attitudes disabling participation. Stigma within families and communities associated with abandonment by parents and being disowned by males responsible for pregnancy mean that girls are not supported to access maternity care.	Clara, Dawn’s caregiver, Efe, Fatmata, SH-DC, SH-RC, SH-HCW
Lack of understanding of disability issues	Limited understanding of disability among health workers and community members functioned as a social barrier to accessing care, especially for girls with invisible impairments. Myths regarding the cause of impairments impact on girls’ rights to care for and breastfeed their babies.	Adama’s caregiver, Balu’s caregiver, Fatmata, SH-HCW, SH-RC, SH-DC
Infrastructural limitations	Environmental barriers. Many healthcare facilities are not disability-friendly, and the lack of accessible transport are barriers to healthcare attendance.	SH-HCW, Clara, SH-NGO, SH-NC, SH-DC, SH-NC

DC, district council; HWC, healthcare worker; NGO, non-governmental organisation; RC, religious council; SH, stakeholder.

### Policy/practice disconnect

Interviewees highlighted multiple times that they are expected to pay for services (registration) and drugs tagged as not a free healthcare drug. Fatmata, who has a learning disability, said her caregiver bought drugs for blood enhancement after the diagnosis that she is pale, needing a ‘blood boost’:

“Yes we were buying medicines, like blood medicines, the one that is in that packet they brought it, then I started drinking it because they said that I looked pale.” (Fatmata)

SH-RC who happens to be one of the stakeholders monitoring the FHCI, explained his belief that the government is making the provisions but those implementing them are not effective in delivering the required services:

“The authorities may do the needful for it to be a real free health care but it depends on those that are to implement it, how effective it is. I would advise that there is still more to be done in monitoring.” (SH-RC)

Representing the views of NCPD, SH-NC emphasised that the actual activities of services rendered to people with disabilities do not match with policies and proclamations of the government:

“First of all, the free health care for disabled girls and disabled persons is one we take with a mixed feeling. First, of course, every woman must be part of the FHC initiative, and it is a requirement in the Disability Act Section 11 that every person with a disability has the right to free medical care. It is there in theory, but what is obtained or practised is a bit different. When they go to these facilities, they are faced with lots of discrimination, the nurses. Some of the nurses are so unprofessional to the point that they tend to discriminate against these women or girls with disabilities. That makes it almost impossible for them to visit these centres for the second time. That is why we say it’s a mixed feeling. First, there are laws, and there are policies that say they should go there, but again, the stigma they face when they go there serves as a deterrent to them.” (SH-NC)

“Well, my view is, by Government nomenclature, it is supposed to be free and they are supposed to be given care without money, but that is not what I am seeing. On the contrary, they (people with disabilities) are almost always abandoned. When you go to the free health care facility expecting that you should be given fair treatment, sometimes it does not happen that way. When you go there, they ask first to register (fee). Secondly, they ask you to give money to undertake all other proceedings. To me, it is no free health care we are seeing, especially for the disabled women.” (SH-DC)

Regarding the perceptions of current training of healthcare workers in disability issues;

“Special training is needed for medical personnel on dealing with persons with disability. They are limited in know-how about disability issues. People say, because you are disabled, you should not enjoy sex, have a family or get pregnant. Those myths affect people with disabilities. Health care professionals should advise who should and who should not, not the layman*.”* (SH-NC)

When asked if there was any training for medical practitioners on how to care for people with disabilities, SH-NC reported asking a health professional who said she had not been offered training.

Given the Basic Package of Essential Health Services (BFUHS) 2015–2020 (14), the identification of disability, especially among children, is expected to happen at the community level in primary healthcare facilities. However, SH-HCW, matron at a main referral hospital, confirmed that this is not actioned and that PHUs are not sufficiently equipped or staff sufficiently trained to diagnose most impairments, with particular issues for impairments that are less visible, such as learning disabilities.

It was also a telling observation that the hospital registers provided by the ministry of health used in antenatal clinics had no mechanism for capturing impairments, making data collection about rates of disability among pregnant women difficult to assess. When asked why this was the case, SH-HCW responded:

“Well, it’s just unfortunate that it is not included in the register but in her book (exercise book carried by a woman), it will be indicated.”

### Social stigmatisation of girls with disabilities

Some parents we interviewed expressed disappointment that their child had an impairment. Others see the extra care needed for the child as a burden, and if such a child gets pregnant, they will have an additional burden, which is frustrating. When asked what her feelings were about her child with disability getting pregnant, Dawn’s caregiver said;

“Well, I did not feel good because she is not well, she can’t talk properly. Even about her laundry, I do it for her. I do everything for her. She cannot do … Anything on her own, so I didn’t feel good.” (Dawn’s caregiver)

Such feelings led some caregivers to abandon pregnant girls with disabilities. Another abandonment comes from the men and boys who impregnate these girls. Five out of six of the men responsible for the girls’ pregnancies had not taken responsibility. Even for those who do accept responsibility legally, in reality, most girls are abandoned. For example, one man who was taken to the police and accepted responsibility relocated to an unknown community as soon as he was granted bail from the police cells:

“We asked the boy, but at first he denied it, so we went to the FSU (Family Support Unit) and reported. They arrested the boy, and he was imprisoned for four days. That was when he agreed. But since his aunt came here and said that they will go and discuss the matter and get back to me, it has been months now, up till now I haven’t seen them.” (Dawn’s caregiver)

She told us

“Let me say about 90% of them, the men don’t take responsibility, if they are challenged. If the men don’t take responsibility for the pregnancy, for them to even take care of the children born is a problem, you know.” (Dawn’s caregiver)

The impact of male abandonment was also highlighted by SH-DC:

“Most likely, these disabled adolescents are always abandoned, men are not happy to associate themselves with them after they have been impregnated, and some men will even ignore their responsibilities because they do not want to associate themselves with them. And many times I have seen them begging on the street for their survival with their children.” *(*SH-DC)

SH-RC expressed concerns about stigma and abuse within families:

“What is sad about this is that their caregivers are abusing most of them. That is the fact, they are stigmatised, maltreated and they say “look at you, you don’t feel sorry for yourself, you got yourself impregnated.” so it’s almost impossible for them to survive and most times because of the pains they go through during the pregnancy, it leads to some traumatic event which might affect the child.” (SH-RC)

Another major source of social stigma which was highlighted by participants was the experience on public transport:

“But public transport is very challenging, maybe someone may say to them don’t touch me with your feet ooh things like that*…”* (SH-HCW)

### Lack of understanding of disability issues

Participants’ experiences revealed that limited understanding of disability among health workers and community members functioned as a key social barrier to accessing care. Despite four of the six girls participating in this study having a learning disability, interviews with the senior health stakeholder and a review of the antenatal clinic register at the referral hospital revealed that there is no dedicated column or mechanism within the register to record disability status. As a result, non-visible impairments such as learning disabilities are not routinely documented. This lack of systematic recording means that girls must disclose their disability at each antenatal visit, often to different healthcare providers. Participants described this repeated disclosure as both frustrating and embarrassing:

“… like when we came today and everyone was scolding her, things like that, she will be shy and won’t even enter because she wouldn’t want to mingle with them, since she knows …. that she is a different person and she won’t be treated like how normal people are treated.” (Adama’s caregiver).

The field researcher observed that Adama could not join or stay in the queue for antenatal care with other patients, and her constant movement angered both the nurses and patients, who scolded her until the intervention of her caregiver, who had to tell them each time of her impairment.

While breastfeeding is widely recognised for its significant preventative health benefits for both mothers and infants, this study found that none of the girls with learning disabilities interviewed were allowed to breastfeed their babies. The girls did not know why they were forbidden to breastfeed, but their caregivers gave them reasons; Balu’s caregiver said she believed the mental illness will be transmitted to the baby through breastfeeding, as she has been warned by the elders of the family not to allow Balu to breastfeed:

“Her uncle even said that since she has delivered, she should not breastfeed the baby because she is mentally unstable*.*” (Balu’s caregiver)

When asked if Adama was breastfeeding her baby, Adama’s caregiver responded:*’*

“Aa no, because she is not mentally normal, her aunt said that she should not breastfeed her child. Of course, you can even see the child’s physical appearance.” (Adama’s caregiver)

This myth is given credence by some healthcare service providers who do not encourage women with disabilities to breastfeed but suggest baby supplements. For example, Adama’s caregiver brought Adama’s baby to the hospital because it was weak and refused to take in a cheap baby supplement donated by community members who volunteered to support with baby food since she was not allowed to breastfeed. Instead of advising the caregiver to breastfeed the baby, the nurse, perhaps influenced by prevailing community beliefs and supply constraints, supported the use of a more expensive formula rather than promoting breastfeeding, highlighting gaps in training on disability-inclusive maternal care.

“I am in the same house as her, I sometimes assist her because her aunt is not always at home. They used to buy NaN (baby formula) for the child, but when she came here today, they talked to her saying that NaN is not good for the child and that she should buy SMA so…. She was even wondering where her Aunty would get that money from…… because her aunty thinks that if she breastfeeds the child, the child will be like the mother*.”* (Adama’s caregiver)

For girls with learning disabilities, we found that major decisions impacting their lives are taken by caregivers or the wider community, as in the case of Fatmata, who has a spinal deformity and learning disability. She had an arranged marriage after her aunt entered into an agreement with another family who had a son with Down syndrome. The family wanted an offspring from their son and were desperate to find a girl who would not mind their son’s condition. When they found Fatmata, who could not give consent, her parents were contacted, and they agreed to the marriage, leaving her with no choice but to accept the marriage, which she believed would make her parents happy. When asked if she was happy with the arranged marriage, Fatmata responded:

“Because I did not want to get married, but when I saw how happy my people were, and they went with me to stay …because …. they wanted the marriage to happen in the village*.”* (Fatmata)

It was observed that traditional beliefs inform attitudes towards people with disabilities, including assumptions, misconceptions, traditional or religious beliefs and beliefs about the natural and supernatural worlds. From the interviews with stakeholders, there is a pervasive perception that people with disabilities are generally daring, disrespectful or easily angered. Some perceive them as witches who invoke difficulties for those who empathise with them, and others believe that their impairment is a result of a curse sanctioned by God.

“Disabled life is very challenging, that is why you find out that they are very mouthy and disrespectful… Because they have to fight for themselves.*”* (SH-HCW)

“People would tend to give some unacceptable treatment to them because of their behaviour. When I complained of ill-treatment meted out on a pregnant disabled woman in a PHU, my daughter, who is a trained health care provider, said …. Father, but those people, they are hard to deal with anyway.” (SH*-*RC)

SH-DC observed that people are afraid to give money to beggars with disabilities because it brings hardship to the giver:

“They are always abandoned. In fact, in my community, some people even feel that they are witches. When people see a disabled person with children on the street begging, they will ask them if they are not from a family, or how they got impregnated without a man taking responsibility. Sometimes people think that giving them money will bring problems to their finances … Some people think if you give money to the disabled you will be harming yourself as they will manipulate the money so that you will not get money again, and you end up suffering as they are suffering also, People have these stereotypical thoughts about the disabled*.”* (SH-DC)

### Infrastructural limitations

Despite the Disability Act 2011 which mandates that all public buildings should be accessible to people with disabilities by 2016, the main district government hospital where the study was conducted was built during the colonial period, and no major refurbishment has been done to enact the policy;

“The time this hospital was built, they never thought of it. When a disabled person comes, it’s usually straining, we will have to carry the wheelchair, carry her for a checkup and carry her back outside because the ones … who built the hospital didn’t think about that. It’s not disability-friendly at all.” (SH-HCW)

“The facility in itself is not conducive for a disabled person, like I was saying, if someone comes in a wheelchair, except we will have to lift them.” (SH-HCW)

“Yes it’s easy to access, for me with the crutches, I can easily walk in but for those using wheelchairs, it could be very difficult. One of my friends on a wheelchair found it difficult to get into the facility.” (Clara)

SH-NGO reported:

“It all boils down to accessibility, and it is enshrined in the disability act, I think section 20 of 2023, which says every public facility should be accessible to persons with disability. So accessibility goes beyond a ramp, railing or the door or the space in the toilet, but even the route leading to a particular health centre has to be accessible to persons with disability. In addition, I will say most of the health facilities built these days have some amount of disability friendliness. For example, there are ramps, some of the tiles are not smooth but others are, and walking with crutches, they might fall. To rate it, the accessibility at these centres is just about 40%.” (SH-NGO)

Access to healthcare for people with disabilities will never be possible if they cannot get to the health centres in the first place. All the girls and caregivers interviewed said the taxi motorbikes, the available commercial transport system in the study site, are not safe as they take two pillion without helmets. However, they are fast and take one to the spot requested, which is helpful for people with disabilities. An additional benefit is that no other passengers can stigmatise the disabled passenger, as there is no need to share a seat with someone else. SHNC*,* referring to the NCPD, said:

“Basically as a nation, we are struggling with transportation, no customised or specialised public transport for persons with disability, or infrastructures for public transportation… the facilities that need to be provided by the Central Government, where reserved seats are marked for the disabled. They are not there, not even the available bus stops have signs or places allotted for persons with disability. It is just not there exclusively for persons with disabilities*.”* (SH-NC)

“There are lots of disabled people struggling even for them to get a wheelchair.” (SH-DC)

“Nevertheless, 99% of public transport drivers will not give someone assistance; they will not even consider the disabled. This is the main problem.” (SH-RC)

“Some public transportation doesn’t even consider people with disabilities. One passenger said to me: I would rather not have a child than have one that uses a wheelchair.” (SH-NC)

## Discussion

Interpreted through the social model of disability, the findings of this qualitative study demonstrate that the primary challenges faced by pregnant adolescent girls with disabilities in Sierra Leone stem not from their impairments, but from socially and structurally produced barriers. These findings suggest that current maternal and adolescent health strategies, while progressive in intent, are insufficiently equipped to address the intersecting vulnerabilities associated with disability, adolescence and gender. Stigma, inaccessible infrastructure, negative provider attitudes and weak institutional support systems collectively shaped participants’ experiences of pregnancy and healthcare access. These findings reinforce critiques of individualised or medicalised understandings of disability in sexual and reproductive health research. Findings revealed four interconnected themes: weak implementation of disability-responsive policies, social stigma and exclusion, limited understanding of disability (especially intellectual impairments) and infrastructural barriers. Many girls’ learning disabilities led to harmful labels and exclusion from decisions like breastfeeding and marriage. Stigma and discrimination were common in public spaces and within families, sometimes resulting in abandonment. Physical inaccessibility of health facilities and transport further restricted care. Finally, despite initiatives like the FHCI and Disability Act, some girls still paid for services, and healthcare providers often lacked disability-specific training, leading to inadequate support.

While the Government of Sierra Leone has demonstrated a strong commitment to maternal and adolescent health through initiatives like the FHCI and the Radical Inclusion Policy, our findings suggest that certain groups—particularly adolescent girls with learning or physical disabilities—continue to face systemic barriers. For instance, while Sierra Leone’s 2011 Persons with Disability Act affirms the rights of persons with disabilities, it does not explicitly name intellectual impairments, contributing to challenges in recognition, diagnosis and service provision for this subgroup. Alignment with international conventions such as the Convention on the Rights of Persons with Disabilities (CRPD), which defines disability more broadly, would help ensure that all forms of disability are adequately acknowledged and supported.

By definition, according to CRPD^15^ which Sierra Leone ratified in 2010, disability is defined as ‘long-term physical, mental, intellectual, or sensory impairments which in interaction with various barriers may hinder [a person’s] full and effective participation in society on an equal basis with others.’ (Article 1). However, Sierra Leone’s 2011 Person with Disability Act (11) defines ‘disability’ as ‘a physical, sensory, mental, or other impairment which has a substantial long-term adverse effect on a person’s ability to carry out normal day-to-day activities’, adhering to a more medical model of disability (in which a person’s impairment is seen to be the thing which disables them, rather than by barriers in society). It also omits an overt reference to intellectual impairments. This means that the process by which impairments are identified and diagnosed in Sierra Leone, including how people are formally registered, does not align with CRPD since disability is not a criterion for one to be classed as disabled.

According to the Basic Package of Essential Health Services 2015–2020^16^ the identification of disability (especially among children) is expected to happen at the community level in primary healthcare facilities. However, interviewees suggested that there is no clear pathway for identifying and diagnosing intellectual impairments. These factors mean that girls with impairments frequently are not diagnosed because of the lack of knowledge about their condition, lack of professional support around learning disabilities in their communities or because of prevailing traditional beliefs. This gap is often filled by using traditional medicine to find the cause of disability^17^ ^18^ and to ‘treat’ the learning disability, and yet none of the participants mentioned traditional healers identifying and then referring persons with learning disabilities to health professionals.

Breastfeeding is one of the most effective ways to ensure a child’s health and survival. Non-breastfed infants in low-resource settings like Sierra Leone face a 13 times higher risk of death from preventable causes (eg, diarrhoea, pneumonia, malnutrition) compared with exclusively breastfed infants.^18^ In sub-Saharan Africa, initiation of breastfeeding within the first hour may prevent about 20% of neonatal deaths, and optimal breastfeeding practices (early initiation+exclusive breastfeeding) have the potential to prevent approximately 12% of all under-5 deaths.^19^ Despite this, only 54% of the babies are exclusively breastfed for up to 6 months, while only 30% continue to be breastfed until 2 years.^20^ Most participants in this study felt that adolescent girls with learning disabilities were not encouraged to breastfeed, with a common belief that intellectual impairments can be passed from mother to baby through breast milk. This view was reinforced by health workers, for example, when a nurse recommended a particular brand of formula milk instead of promoting breastfeeding, despite the Government Sierra Leone approving a bill to regulate the marketing of breastmilk substitutes to improve the nutrition of infants and young children.^21^ With the recognition that breastfeeding is pivotal to bonding, communication and child development^22^ caregivers felt that breastfeeding should not be left to mothering adolescents with learning disabilities, as this may impact on the normal development of the baby. From the limited data available, it appeared that the positive benefits of breastfeeding for infant survival and lifelong health were overlooked or seen as secondary compared with the perceived risk to the baby of acquiring a learning disability through breastmilk, and it was notable that the opinions of these young mothers was not sought or taken into account when infant feeding decisions were made. A major recommendation of this study is health worker education and community sensitisation to break down these misconceptions, and that mothers with learning disabilities are supported at both the healthcare level and community level to breastfeed their babies.

It was clear from discussions with caregivers and girls themselves that girls with both physical and learning disabilities are often not given a voice when it comes to decisions about other important life issues, such as marriage. Usually, this comes from a place of concern about a child’s future and long-term care, as well as the desire for grandchildren. From our sample, two of the girls with learning disabilities had been forced into a marriage with babies removed from their care to the care of the grandparents. The stigma surrounding disability is indeed deeply entrenched in Sierra Leone, with a widely held belief that it is caused by a parent’s sin or the work of the devil, or an attack from a ‘spirit husband’ and other forms of witchcraft, or that the disability is contagious.^23 24^ In Sierra Leone, people with physical disabilities are often called ‘crippled’, or ‘die futahn’ (dead feet or dead hands) in Krio.^25^ Children with severe impairments are referred to as ‘debulpikin’, which means ‘devil’ or ‘demon child’ and people with amputations are often called ‘one leg’.^26^ When a baby is born with an impairment, families sometimes leave their babies in the forests, believing that the child must be returned to the demon spirits where they came from. Others hide them from public view or abandon them altogether. This often leads to children being excluded from village life as others are fearful of ‘catching’ the impairment.^17^

Barriers to access healthcare for pregnant or mothering adolescents are commonly associated with stigma and negative societal and individual attitudes, including from healthcare providers, and often result from a skills gap, inadequate infrastructure, materials, equipment, policies and programming which are unable to effectively address the needs of persons with impairments, rather than with the impairments themselves.^26^ There is a common belief that those with disabilities should not have sexual relationships or become mothers, impacting on their willingness to access sexual and maternity healthcare.^27 28^ In a study in Ghana, women with disabilities encountered direct discrimination from maternal health nurses, with one woman being referred to as a pregnant cripple, while another was refused physical assistance.^29^ Women with impairments may deliberately avoid healthcare appointments due to experiencing or desiring to avoid negative attitudes from healthcare providers.^29 30^

Adolescent girls who get pregnant are often disowned by their parents, who believe they wasted their money educating daughters who now have no prospects.^28^ The government of Sierra Leone’s Radical Inclusion Policy^31^ attempts to address this lack of educational opportunities for disabled children and pregnant girls, but in our experience, the policy is poorly understood. Within our 2YoungLives mentoring, we have piloted a workshop to try to create an understanding with school principals and teachers and employ deliberate community engagement to help remove the stigma associated with disability and/or pregnancy and redress the lack of opportunity for these vulnerable children. These workshops have resulted in significant shifts in attitudes among teachers and school leaders, opening opportunities for reintegration into school for young mothers with and without disabilities, which the policy alone had not achieved. It is recommended that these workshops are mainstreamed into teachers’ in-service training. Pregnancy is seen to be ‘the ultimate shame’,^32^ and this shame is exacerbated when the teenager is living with a disability. This plays out in narratives of blaming and shaming^33^; one of the girls with learning disabilities who we interviewed disclosed that she was sent away from Freetown, the capital, to the study district so that people in the community would not know about her pregnancy because she was violated by their landlord, whom the aunt wanted to protect from going to jail. People with disabilities are known to be at an increased risk of experiencing violence and abuse, and this risk is further elevated for girls and women^34^ who also face higher rates of homelessness, discrimination and crime, and are often forced to raise children with little to no support.^35^

Social exclusion refers to a process in which a person or group cannot participate effectively in mainstream society due to poor accessibility and mobility, restricting socio-economic participation, which ultimately affects health, life quality, cohesion and equity. Two main infrastructural limitations were identified within this study: the lack of access to suitable transport and the physical barriers experienced during hospital visits. The Disability Act 2011 (10) mandated that all public buildings be disability-friendly by 2016, giving time for suitable adaptations to be made. However, when accessing hospital buildings for antenatal clinics, women with physical disabilities still experience several access barriers, including the limited availability of ramps, rails and wide doors.^8^ Section 25 (1) of the Act states that operators of public service vehicles shall adapt such vehicles to convey persons with disability, in such manner as may be specified by the Commission. Despite this law, the challenges people with disabilities encounter in their routine lives are far greater than those encountered by their able-bodied counterparts. Transport is an essential aspect of accessing healthcare, yet people with disabilities experience restrictions from the lack of customised vehicles, including ambulances, with public transport having no designated space and the lack of signage indicating designated spaces at bus stops and parks. An individual using a wheelchair, crutches or other gadgets to aid their movement who wants to board either a taxi, poda poda (taxi vans) or bus, which are the popular modes of transportation in Sierra Leone, has to rely on the mercy of the driver, conductor or other passengers to help them to enter the vehicle, which can be embarrassing and degrading.

Government policies recognise and emphasise a commitment to the right to health for people with disabilities. The 2011 Disability Act explicitly identifies the right of people with disabilities to free medical services in public health institutions, and the FHCI, launched by the Government in 2010, now also covers this group. Sierra Leone briefly received the largest per-capita aid donations globally. ^36^ Regardless of that significant investment in the postwar re-establishment of the health system, Sierra Leone’s health outcomes languished at the bottom of many health index tables, being among the ten worst-performing countries when it comes to neonatal mortality, infant and under-5 mortality, stillbirth and child death rates.^37^ The maternal mortality rate, once the worst in the world, has improved recently, with a reduction to 354 per 100 000 live births in 2023;^37^ however, structural improvements that could bring further reductions have been slow.^38^ The country suffered a devastating Ebola virus disease outbreak between 2014 and 2016, in which at least 4000 citizens and 221 health workers died.^39^ The introduction of the FHCI marked significant changes; the promise of free healthcare attracted patients in much greater numbers, and while the initial spike in healthcare uptake did not last, the demand for health facilities and hospitals (as opposed to traditional healers and other informal care providers) has remained at a much higher level since 2010.^40^

Within the policy landscape in Sierra Leone, there is provision for ensuring that the health needs of people with disabilities are recognised and addressed^10 41^ and are entitled to free healthcare in public healthcare facilities. However, access to free healthcare for people with disabilities has remained unclear^42^ with vulnerable citizens reporting about paying for drugs and services meant to be free for their group. The drivers for these informal charges are not hard to find; in 2016, a report published by the Ministry of Health and Sanitation^43^ revealed that only half of its health workforce was on the payroll and out of the 9120 unsalaried health workers identified in the payroll audit, approximately 40% were health professionals providing patient services, primarily in the lower-skilled cadres. A 2016 study found that as many as 96% of households paid for supposedly free services^44^ suggesting that charging for care is widespread and seemingly practised by salaried and unsalaried workers alike. If Sierra Leone were to introduce greater monitoring and oversight of its health workers to curtail informal charging, the question remains: how could unsalaried workers cope without pay for prolonged periods? Health service provision in remote and rural areas would likely be much reduced without the contribution of unsalaried health workers. However, their coping mechanism of informal charges might also be one of the barriers to effectively implementing Sierra Leone’s FHCI.

With only two psychiatrists and 19 mental health nurses, Sierra Leone has a minimal ability to diagnose and treat learning conditions. Further, an overall poor understanding of learning disabilities and limited disability training and education in the health workforce have contributed to high levels of stigma toward patients with disabilities.^45^ Health professionals need to be equipped with the necessary knowledge and skills to understand and address the needs and challenges faced by these patients. Such training may encompass disability awareness, communication strategies, providing culturally competent care and ensuring accessible health facilities. Well-trained and motivated health workers are the bedrock for a strong and functioning healthcare system in Sierra Leone and more compassionate and effective care of pregnant adolescents with disabilities, their children and caregivers.

### Strengths and limitations

This study is among the first to explore the perinatal experiences of adolescent girls living with disabilities in Sierra Leone, including those with intellectual impairments who are rarely represented in research. The inclusion of caregivers and stakeholders provided triangulated perspectives on barriers to care. The study is limited by its small sample size and single-district setting, which may limit transferability to other regions, particularly rural areas. However, all eligible girls within the study site were included, offering depth and contextual richness. As with all qualitative research, findings are context-specific, but they provide important insights to inform policy and practice in similar settings.

### Implications for policy and practice

Sierra Leone’s Radical Inclusion Policy aligns with broader regional commitments to inclusive education and health equity across sub-Saharan Africa. However, evidence from Ghana and other countries in the region suggests that progressive inclusion frameworks often struggle at the implementation level, particularly for women and girls with disabilities accessing sexual and reproductive health services. Multiple studies in sub-Saharan Africa similarly document stigma, provider discrimination, inaccessible facilities and limited disability-specific training as persistent barriers, indicating that Sierra Leone’s challenges are not unique but reflect regional patterns of policy–practice disconnect [].^2946^,^48^ This study indicates that the primary policy challenge in Sierra Leone is not the absence of disability-inclusive legislation, but weak implementation, monitoring and accountability. Despite the FHCI and the Persons with Disability Act, informal charging, inaccessible facilities and discriminatory practices persist, undermining policy intent. Our findings highlight an urgent need for disability-inclusive training within the maternal health workforce, particularly around intellectual impairments, communication, consent and infant feeding. Without such training, health workers may unintentionally reinforce harmful community myths, as seen in discouragement of breastfeeding among mothers with learning disabilities. The absence of disability indicators within many registers renders pregnant adolescents with disabilities effectively invisible within routine health data, limiting planning, resource allocation and accountability. Integrating and appropriately recording disability identifiers into maternity records is a low-cost, high-impact intervention.

The barriers to accessing healthcare for girls with impairments during pregnancy and in the postnatal period result not from the impairments themselves, but from a range of social, attitudinal and structural barriers. A lack of knowledge about disability issues by caregivers and healthcare providers, that the FHCI has not been free in reality, and that old health infrastructures are not disabled-friendly or easy to access on public transport. The community myths about disability lead to stigma and neglect by relatives and community members, and lead to girls being prevented from making decisions on important choices such as infant feeding and marriage. The government has made good laws and policies that ensure the alleviation of barriers to healthcare for people with disabilities, however more needs to be done in the implementation of policies and training of healthcare providers on disability. Addressing these barriers requires coordinated action across health, education and social protection sectors, reflecting the intersecting nature of exclusion identified in this study.

## Conclusion and recommendations

While significant progress has been made in improving maternal health and disability rights in Sierra Leone, it is imperative within families, communities and health services, to reframe the deficit medical model of individual disability to an acknowledgement that it is the social, attitudinal, structural and environmental barriers which disable, and that these barriers can indeed be deconstructed. The findings offer valuable insights for strengthening inclusive health policies and practices, and reaffirm the need for collaborative, cross-sectoral approaches that uphold the dignity and rights of girls and young women with disabilities.

Recommendations drawn from the study findings require targeted, collaborative action from key stakeholders. For policymakers, the definition of disability in Sierra Leone must be widened to embrace the social model of disability, in line with the CRPD, explicitly including intellectual impairment; furthermore, the physical adaptation of existing health facilities must be monitored and publicly reported with a clear execution plan. For healthcare providers and health systems, data on disability must be accurately recorded within routine maternity services, and specific training modules must be implemented to equip staff to provide inclusive care and debunk harmful clinical myths, such as the ‘transmission’ of intellectual impairments through breastmilk. Finally, for community leaders and educators, targeted sensitisation initiatives must be rolled out to dismantle social stigma and support mothers to breastfeed, alongside mainstreaming radical inclusion workshops into teachers’ in-service training to promote school attendance for mothering adolescents with disabilities.

## Data Availability

Data are available upon reasonable request.

## References

[1] World Health Organization (2025). Trends in Maternal Mortality Estimates 2000 to 2023: Estimates by WHO, UNICEF, UNFPA, World Bank Group and UNDESA/Population Division.

[2] Ministry of Health and Sanitation, Sierra Leone (2022). National health sector strategic plan 2021–2025.

[3] UNICEF (2024). Situation analysis of children and adolescents in Sierra Leone.

[4] UNICEF (2024). Maternal and newborn health disparities, Sierra Leone.

[5] Fernandez Turienzo C, Kamara M, November L (2024). A community-based mentoring scheme for pregnant and parenting adolescents in Sierra Leone: Protocol for a hybrid pilot cluster randomised controlled trial. PLoS One.

[6] Fernandez Turienzo C, November L, Kamara M (2025). Community-based mentoring to reduce maternal and perinatal mortality in adolescent pregnancies in Sierra Leone (2YoungLives): a pilot cluster-randomised controlled trial. The Lancet.

[7] Statistics Sierra Leone, World Bank and Government of Sierra Leone (2019). Sierra leone integrated household survey (slihs) report 2018.

[8] Magnusson L, Kebbie I, Jerwanska V (2022). Access to health and rehabilitation services for persons with disabilities in Sierra Leone - focus group discussions with stakeholders. BMC Health Serv Res.

[9] Oliver M (2013). The social model of disability: thirty years on. Disabil Soc.

[10] Government of Sierra Leone (2011). Sierra Leone Disability Act 2011.

[11] CRIBS Sierra Leone CRIBS Sierra Leone.

[12] Tong A, Sainsbury P, Craig J (2007). Consolidated criteria for reporting qualitative research (COREQ): a 32-item checklist for interviews and focus groups. Int J Qual Health Care.

[13] Braun V, Clarke V (2006). Using thematic analysis in psychology. Qual Res Psychol.

[14] November L, Kamara M, Kamara P (2024). Meaningful community engagement and involvement in global health and research: “Changing mindsets with a million conversations” in Sierra Leone. J Glob Health.

[15] United Nations General Assembly (2007). Convention on the rights of persons with disabilities: resolution adopted by the general assembly, 24 january 2007, a/res/61/106.

[16] Sierra Leone Ministry of Health (2015). Sierra-leone-essential-hs-package-draft-final. https://mohs2017.files.wordpress.com/2017/06/gosl_2015_basic-package-of-essential-health-services-2015-2020.pdf.

[17] Berghs M, Dos Santos–Zingale M (2011). A Comparative Analysis: Everyday Experiences of Disability in Sierra Leone. Afr Today.

[18] Victora CG, Bahl R, Barros AJD (2016). Breastfeeding in the 21st century: epidemiology, mechanisms, and lifelong effect. The Lancet.

[19] Zhao M, Wu H, Liang Y (2020). Breastfeeding and Mortality Under 2 Years of Age in Sub-Saharan Africa. Pediatrics.

[20] UNICEF, WHO (2021). Only 54% of babies are exclusively breastfed up to 6 months, and just 30% are breastfed to 2 years in Sierra Leone.

[21] Scaling Up Nutrition (2021). Sierra Leone passes ground-breaking law to protect breastfeeding, 6 August. Scaling Up Nutrition.

[22] Papp LM (2014). Longitudinal associations between breastfeeding and observed mother-child interaction qualities in early childhood. *Child Care Health Dev*.

[23] Njelesani J, Hashemi G, Cameron C (2018). From the day they are born: a qualitative study exploring violence against children with disabilities in West Africa. BMC Public Health.

[24] Groce N, McGeown JW (2013). Wealth and disability: reinterpretation of a folk belief in contemporary urban africa (june 3, 2013). leonard cheshire disability and inclusive development centre working paper series: no.30. https://ssrn.com/abstract=3398227.

[25] Andregård E, Magnusson L (2017). Experiences of attitudes in Sierra Leone from the perspective of people with poliomyelitis and amputations using orthotics and prosthetics. Disabil Rehabil.

[26] Montrose (2020). Access to Health Services of People Living with Disability: A Literature Review as Part of MELR Research Part 1: Understanding Access to Free Health Care Initiative (FHCI) for Persons Living with Disabilities and Adolescent-Friendly Services Protocol as Part of Monitoring, Evaluation, Learning and Review for Saving Lives in Sierra Leone (SLiSL) Programme.

[27] UNGEI and Sightsavers (2021). Leave no girl with a disability behind: voices from girls with disabilities in sierra leone.

[28] Carew MT, Dhingra S, Bash-Taqi R (2024). “These attitudes are a pressure”: women with disabilities’ perceptions of how stigma shapes their sexual health choices. *Culture, Health & Sexuality*.

[29] Ganle JK, Baatiema L, Quansah R (2020). Barriers facing persons with disability in accessing sexual and reproductive health services in sub-Saharan Africa: A systematic review. PLoS One.

[30] Ganle JK, Otupiri E, Obeng B (2016). Challenges Women with Disability Face in Accessing and Using Maternal Healthcare Services in Ghana: A Qualitative Study. PLoS One.

[31] Ministry of Basic and Senior Secondary Education (2021). National Policy on Radical Inclusion in Schools.

[32] Peyton N, Just say no? Sierra Leone tests new ways to cut teen pregnancy (2019). Thomson reuters foundation news [internet]. https://news.trust.org/item/20190905160253-rkzh7/.

[33] November L, Sandall J (2018). “Just because she’s young, it doesn’t mean she has to die”: exploring the contributing factors to high maternal mortality in adolescents in Eastern Freetown; a qualitative study. Reprod Health.

[34] Fang Z, Cerna-Turoff I, Zhang C (2022). Global estimates of violence against children with disabilities: an updated systematic review and meta-analysis. *The Lancet Child & Adolescent Health*.

[35] National Democratic Institute (2023). Amplifying disability rights through national debate in sierra leone. https://www.ndi.org/our-stories/amplifying-disability-rights-through-national-debate-sierra-leone.

[36] Barr A, Garrett L, Marten R (2019). Health sector fragmentation: three examples from Sierra Leone 16 studies in Human Society 1605 policy and administration. Glob Health.

[37] UN Inter-agency Group for Child Mortality Estimation (2023). CHILD MORTALITY AND STILLBIRTH ESTIMATES.

[38] Integrated African Health Observatory, WHO (2023). Maternal mortality: The urgency of a systemic and multisectoral approach in mitigating maternal deaths in Africa.

[39] Elston JWT, Danis K, Gray N (2019). Maternal health after Ebola: unmet needs and barriers to healthcare in rural Sierra Leone. Health Policy Plan.

[40] Raven J, Wurie H, Witter S (2018). Health workers’ experiences of coping with the Ebola epidemic in Sierra Leone’s health system: a qualitative study. BMC Health Serv Res.

[41] Edoka I, Ensor T, McPake B (2016). Free health care for under-fives, expectant and recent mothers? Evaluating the impact of Sierra Leone’s free health care initiative. Health Econ Rev.

[42] Government of Sierra Leone, Ministry of Health and Sanitation (2016). Human Resources for Health, Sierra Leone Country Profile.

[43] Witter S, Brikci N, Harris T (2018). The free healthcare initiative in Sierra Leone: Evaluating a health system reform, 2010-2015. Int J Health Plann Manage.

[44] Brooks A, Herrick C (2019). Bringing relational comparison into development studies: Global health volunteers’ experiences of Sierra Leone. *Progress in Development Studies*.

[45] Jofre-Bonet M, Kamara J, Mesnard A (2021). Corruption and Health Insurance for the Informal Sector in Sierra Leone.

[46] Kvam MH, Loeb M, Mitra S (2015). The impact of disability on maternal health care utilization in sub-Saharan Africa. Health Econ.

[47] Ahumuza SE, Matovu JKB, Ddamulira JB (2014). Challenges in accessing sexual and reproductive health services by people with physical disabilities in Kampala, Uganda. Reprod Health.

[48] Shakespeare T, Iezzoni LI, Groce NE (2009). Disability and the training of health professionals. The Lancet.

