# Supplementary material for: Barriers to healthcare access during pregnancy and after birth for adolescent girls living with disability in Sierra Leone: a qualitative study
Source: BMJ Open. 2026 Jun 2;16(6):e109084. doi: 10.1136/bmjopen-2025-109084 (PMC13239463; doi:10.1136/bmjopen-2025-109084)
Supplement: Supplementary file 1 [file bmjopen-2025-109084.reviewer_comments.pdf]

PEER REVIEW HISTORY

BMJ Open publishes all reviews undertaken for accepted manuscripts. Reviewers are asked to complete a checklist review form and are provided with free text boxes to elaborate on their assessment. These free text comments are reproduced below.

ARTICLE DETAILS

Title (Provisional)

Barriers to health care access during pregnancy and after birth for adolescent girls living with disability in Sierra Leone: a qualitative study

Authors

Kamara, Philemon; Kamara, Mangenda; November, Lucy; Sandall, Jane; Williams, Prince T; Wurie, Haja; Fernandez Turienzo, Cristina

---

VERSION 1 - REVIEW

**Reviewer**	**1**
**Name**	**Campero, Lourdes**
**Affiliation**	**National Institute of Public Health, Population Health Research Center**
**Date**	**10-Dec-2025**
**COI**	**None**

This is an important qualitative study addressing a critical gap issue in many countries and the manuscript is clearly written. However, I mention below some aspects and suggestions that could improve its quality.

Methods: Clarify the sampling strategy.

Results: Consider starting the findings from the general to the specific. That is, begin with "Disconnect between policies and practices". Check that all three participant groups (girls, caregivers, stakeholders) are represented within each theme where possible. It is important to better link the findings to the framework. Explicitly strengthen the four outcome themes as manifestations of violations of the fundamental principles of the CRPD (Example: lack of understanding violates respect for inherent dignity...). Add a results summary before discussion.

It would be valuable to add some specific and practical recommendations on breastfeeding. This is a very important topic in the manuscript and could be further developed in the discussion. Some of the important limitations that must be addressed are the small sample,

one district and interview duration. Also discuss how findings might be transferred to other similar districts and give some specific recommendations regarding the main results.

Reviewer 2

Name Saah, Farrukh

Affiliation University of Cape Coast College of Humanities and Legal Studies, Population and Health

Date 02-Feb-2026

COI None

This paper presents a significant contribution to addressing the barriers to health care access for pregnant and parenting disabled adolescent girls in Sierra Leone. However, there are many issues that the authors have to address to improve the quality and rigour of the draft. Here are my comments:

Abstract:

1. The background does not clearly state what the research problem or gap is. Which healthcare barriers are the authors referring to, for which they planned to investigate further, as stated in the objective? Authors should revise.

2. Rather than focusing on ethics in the methods, authors should instead focus on the design, population, data collection tools and procedures, and the data analysis.

3. Change “hindering” to “hinder” in the results to match the other themes’ structure.

4. It is suggested that the conclusion cover the implications of the findings rather than just a summary of the results.

Introduction:

5. There is the most recent data on MMR that puts the figure at 354 per 100,000 in 2023. Authors should check and reconcile with the most recent data.

6. The research problem/gap necessitating the study is not clearly explained. Authors should revise the current draft. Also, include the significance/justification of the study in the introduction.

Methods:

It is suggested that the authors restructure this section, as many things are mixed up.

7. The section should include: Study site/setting description

8. Some details in the population and recruitment are meant for "Data collection". As such, authors should revise the subsection and have separate subsections for "Data collection" and "Data analysis".

Results:

9. While the authors mention creating a "thematic map", one is not included in the text. Include a thematic table/map that presents the themes and their definitions to provide a mind's overview of the findings.

10. I find the themes very descriptive rather than analytical. I suggest the authors revise this section to ensure analytical depth is achieved.

11. I find it odd that no framework/theory was guiding this important qualitative research. This would have underpinned the findings and their analytical depth as well as improved the presentation of this section. Using a framework (e.g., the Social Model of Disability or an Intersectionality Framework) would move the results from descriptive "themes" to a more robust analysis of power dynamics and systemic exclusion. Authors should revise.

Discussion:

12. This section is extensive but lacks discussion of the inferences and implications of the findings for policy and practice. Although the authors do briefly mention a study from Ghana, the discussion could be much broader in comparing Sierra Leone's Radical Inclusion Policy with similar regional health equity frameworks. There is a need to revise the section and include a subsection on the "Strengths and Limitations" of the study.

Conclusion:

13. Authors should include a separate section drawing conclusions and recommendations from the implications of the study findings. The current conclusion is quite general, calling for "increased advocacy". Specific, actionable recommendations for the Ministry of Health regarding the inclusion of disability columns in antenatal registers would add significant value.

VERSION 1 - AUTHOR RESPONSE

Comments from reviewer #1

This is an important qualitative study addressing a critical gap issue in many countries and the manuscript is clearly written. However, I mention below some aspects and suggestions that could improve its quality.

Comment 1: Methods: Clarify the sampling strategy.

Response: *Thank you. We have described the sampling strategy for all groups of participants in the 'participants and recruitment' section (Page 5).*

Comment 2: Results: Consider starting the findings from the general to the specific. That is, begin with "Disconnect between policies and practices". Check that all three participant groups (girls, caregivers, stakeholders) are represented within each theme where possible. It is important to better link the

findings to the framework. Explicitly strengthen the four outcome themes as manifestations of violations of the fundamental principles of the CRPD (Example: lack of understanding violates respect for inherent dignity...). Add a results summary before discussion.

Response: *Thank you. We have re-ordered the themes to move from general to specific in the revised manuscript (pages 8-15) We have also added a summary table of the themes with a brief description and specifying which participants contributed to the theme (page 15).*

Comment 3: It would be valuable to add some specific and practical recommendations on breastfeeding. This is a very important topic in the manuscript and could be further developed in the discussion. Some of the important limitations that must be addressed are the small sample, one district and interview duration. Also discuss how findings might be transferred to other similar districts and give some specific recommendations regarding the main results.

Response: *We have added to the discussion on the breastfeeding issue and have included an explicit recommendation (page 18).*

Comments from reviewer #2

This paper presents a significant contribution to addressing the barriers to health care access for pregnant and parenting disabled adolescent girls in Sierra Leone. However, there are many issues that the authors have to address to improve the quality and rigour of the draft. Here are my comments:

Abstract

Comment 1: The background does not clearly state what the research problem or gap is. Which healthcare barriers are the authors referring to, for which they planned to investigate further, as stated in the objective? Authors should revise.

Response: *Thank you. We have added clarity on this by changing the wording of the Background and objective in the revised manuscript (page 3, abstract).*

Comment 2: Rather than focusing on ethics in the methods, authors should instead focus on the design, population, data collection tools and procedures, and the data analysis.

Response: *Response: Thank you. We have taken the focus off ethics in this section, dealing with it in the 'Data Collection' and Data analysis sections in Methods (pages 6-7).*

Comment 3. Change "hindering" to "hinder" in the results to match the other themes' structure.

Response: *We changed 'hindering' to 'hinders'.*

Comment 4. It is suggested that the conclusion cover the implications of the findings rather than just a summary of the results.

Response: *Thank you. We have added a sentence to the conclusion in the revised manuscript.*

Introduction

Comment 5: There is the most recent data on MMR that puts the figure at 354 per 100,000 in 2023. Authors should check and reconcile with the most recent data.

Response: *Thank you. We have amended the statistic at both references and updated the reference (page 3).*

Comment 6. The research problem/gap necessitating the study is not clearly explained. Authors should revise the current draft. Also, include the significance/justification of the study in the introduction.

Response: *Thank you we have added few sentences to the background to add clarity (page 4).*

Methods:

Comment 7. It is suggested that the authors restructure this section, as many things are mixed up. The section should include: Study site/setting description.

Response: *Thank you. We have simplified and restructured this section (pages 5-6).*

Comment 8. Some details in the population and recruitment are meant for "Data collection". As such, authors should revise the subsection and have separate subsections for "Data collection" and "Data analysis".

Response: *Thank you. We have moved the content on data collection from 'participants and recruitment' to 'data collection' and have split up 'data collection and analysis' into 2 separate sections (pages 6-7).*

Results

Comment 9. While the authors mention creating a "thematic map", one is not included in the text. Include a thematic table/map that presents the themes and their definitions to provide a mind's overview of the findings.

Response: *Thank you. We have added a summary table of the themes at the end of the Results section as also requested by Reviewer 1.*

Comment 10. I find the themes very descriptive rather than analytical. I suggest the authors revise this section to ensure analytical depth is achieved.

Response: *We have reviewed this section in the revised manuscript and believe the analysis of the results is developed in the Discussion (10-16).*

Comment 11. I find it odd that no framework/theory was guiding this important qualitative research. This would have underpinned the findings and their analytical depth as well as improved the presentation of this section. Using a framework (e.g., the Social Model of Disability or an Intersectionality Framework) would move the results from descriptive "themes" to a more robust analysis of power dynamics and systemic exclusion. Authors should revise.

Response: *Thank you for this helpful suggestion. We have reframed the study within the social model of disability framework.*

Discussion

Comment 12. This section is extensive but lacks discussion of the inferences and implications of the findings for policy and practice. Although the authors do briefly mention a study from Ghana, the discussion could be much broader in comparing Sierra Leone's Radical Inclusion Policy with similar regional health equity frameworks. There is a need to revise the section and include a subsection on the "Strengths and Limitations" of the study.

Response: *Thank you. We have substantially revised the Discussion section to strengthen interpretation of findings and explicitly articulate implications for policy and practice. We added a dedicated subsection on policy and practice implications, expanded regional comparisons situating Sierra Leone's Radical Inclusion Policy within broader sub-Saharan African equity frameworks, and included a strengths and limitations subsection (pages 18-22)*

Conclusion:

Comment 13. Authors should include a separate section drawing conclusions and recommendations from the implications of the study findings. The current conclusion is quite general, calling for "increased advocacy". Specific, actionable recommendations for the Ministry of Health regarding the inclusion of disability columns in antenatal registers would add significant value.

Response: *Thank you, we have incorporated a separate conclusions and recommendations section in the revised manuscript (page 23).*

VERSION 2 - REVIEW

**Reviewer**	**1**
**Name**	**Campero, Lourdes**

Affiliation **National Institute of Public Health, Population Health Research Center**

Date **20-Mar-2026**

COI

The authors of this manuscript have taken into account the suggestions I made in the first review of the paper, therefore, I have no further comments to make.

Reviewer **2**

Name **Saah, Farrukh**

Affiliation **University of Cape Coast College of Humanities and Legal Studies, Population and Health**

Date **18-Mar-2026**

COI

I appreciate the authors' thorough and constructive revisions in response to my earlier comments. Across all sections, the manuscript has been strengthened considerably. However, there are still a few issues I suggest they address to improve and finalise the manuscript for publication:

Abstract:

1. Redundancy in Background: The sentence "Across the whole cohort, the program revealed challenges such as neglect by families and stigma within communities and healthcare facilities. However, for girls living with impairments, these risk factors appeared to be exacerbated…" overlaps conceptually with the later statement about barriers and risks. It could be streamlined to avoid repetition.

2. Overstatement of prevalence: "In Sierra Leone, 4.3% of the population lives with a disability, with young adolescents particularly affected…" feels slightly imprecise. The prevalence figure is clear, but the claim that "young adolescents [are] particularly affected" is not quantified. This could be seen as an overgeneralisation unless supported by data in the main text.

3. Methods phrasing: "Participants were invited to participate to face-to-face interviews…" has a grammatical slip ("to participate to"). It should read "invited to face-to-face interviews" or "invited to participate in face-to-face interviews."

4. Design should be "Qualitative method was used in this study"

5. Results section punctuation: The semicolon at the end of the results list ("remaining non-compliant with disability regulations;") is awkward. It should end with a period for consistency.

6. Conclusion scope: While advocacy and training are mentioned, the conclusion could be seen as somewhat generic. It doesn't specify whether these solutions are aimed at policymakers, healthcare providers, or community leaders. This vagueness might weaken the impact.

Background:

7. Sentence structure and flow: Some sentences are long and packed with multiple ideas (e.g., the one beginning "Adolescent girls account for about 20 to 40%…"). Breaking them into shorter sentences would improve readability.

8. Precision of statistics: The range "20 to 40% of all maternal deaths" is quite wide. If this comes from different sources, clarifying why such a broad range exists would strengthen credibility.

9. Minor grammatical slips: "…leading to incorrect advice about breastfeeding. This prompted our desire to conduct this qualitative study…" → "prompted our decision" or "prompted us to conduct" would be smoother.

"…In contrast to medical or individual, the social model…" → should read "In contrast to the medical or individual model…".

10. Redundancy: Disability prevalence (4.3%) is mentioned in both the abstract and background. While not incorrect, it could feel repetitive unless tied to a distinct point in each section.

11. Framing of justification: The justification is present, but the link between the pilot trial results (48% reduction in mortality) and the qualitative study could be made sharper. Right now, the transition feels slightly abrupt—moving from trial outcomes to disability issues without a clear bridge.

Methods:

12. All my comments on this section have been well addressed. It is now logically structured, with clear subsections for setting, recruitment, data collection, and analysis. The restructuring improves readability and methodological transparency.

Findings:

13. "Table 2. Theme summary" should precede the presentation of the detailed thematic findings with a short narrative of what themes were identified before going into details of each theme with their quotes.

14. Typo in the first paragraph of "Social stigmatisation of girls with disabilities" in "disability" spelling

15. Overall, the section remains descriptive, but now sits within a clear framework, and the analytic unpacking is deferred to the Discussion.

Discussion:

16. Some subsections (Strengths and limitations and Conclusion and recommendation) have not been clearly demarcated. They seem to join the starting sentences. Format these appropriately.

17. My comment has been fully addressed. The revised Discussion now has clear analytical inferences, policy/practice implications, regional comparisons, and a strengths/limitations subsection. The section is much stronger and aligns with qualitative reporting standards.

Conclusion:

The comment has been fully addressed. The conclusion now goes beyond general advocacy and provides concrete, actionable recommendations for the Ministry of Health and other stakeholders.

VERSION 2 - AUTHOR RESPONSE

Comments from reviewer #1

Comment 1: The authors of this manuscript have taken into account the suggestions I made in the first review of the paper, therefore, I have no further comments to make.

Response: *Thank you for helping us improve the quality of our paper.*

Comments from reviewer #2

I appreciate the authors' thorough and constructive revisions in response to my earlier comments. Across all sections, the manuscript has been strengthened considerably. However, there are still a few issues I suggest they address to improve and finalise the manuscript for publication

Abstract

Comment 1: Redundancy in Background: The sentence "Across the whole cohort, the program revealed challenges such as neglect by families and stigma within communities and healthcare facilities. However, for girls living with impairments, these risk factors appeared to be exacerbated…" overlaps conceptually with the later statement about barriers and risks. It could be streamlined to avoid repetition.

Response: *We have streamlined this section to avoid repetition: 'In 2017, Lifeline Nehemiah Projects in Sierra Leone launched 2YoungLives, a mentoring initiative for vulnerable pregnant adolescents, including those living with disabilities. Drawing from the social model of disability, we aim to investigate the exacerbated, disabling barriers which prevent these girls from accessing available health care, and develop comprehensive solutions to improve their access'.*

Comment 2: Overstatement of prevalence: "In Sierra Leone, 4.3% of the population lives with a disability, with young adolescents particularly affected…" feels slightly imprecise. The prevalence figure is clear, but the claim that "young adolescents [are] particularly affected" is not quantified. This could be seen as an overgeneralisation unless supported by data in the main text.

Response: *This section has now been amended and streamlined as per previous comment.*

Comment 3. Methods phrasing: "Participants were invited to participate to face-to-face interviews…" has a grammatical slip ("to participate to"). It should read "invited to face-to-face interviews" or "invited to participate in face-to-face interviews."

Response: *We have corrected this in the revised manuscript.*

Comment 4. Design should be "Qualitative method was used in this study"

Response: *We have corrected this in the revised manuscript.*

Comment 5: Results section punctuation: The semicolon at the end of the results list ("remaining non-compliant with disability regulations;") is awkward. It should end with a period for consistency.

Response: *We have corrected this in the revised manuscript.*

Comment 6. Conclusion scope: While advocacy and training are mentioned, the conclusion could be seen as somewhat generic. It doesn't specify whether these solutions are aimed at policymakers, healthcare providers, or community leaders. This vagueness might weaken the impact.

Response: *We have clarified this section, and also the conclusion section of the discussion to clarify that solutions include, for example, the need for policymakers to enforce disability-inclusive policies and infrastructure adaptations, training for healthcare providers to dismantle clinical biases and accurately record disability data, and community leaders to drive local advocacy to break down social stigma.*

Background:

Comment 7. Sentence structure and flow: Some sentences are long and packed with multiple ideas (e.g., the one beginning "Adolescent girls account for about 20 to 40%…"). Breaking them into shorter sentences would improve readability.

Response: *We have amended this to improve readability and flow.*

Comment 8. Precision of statistics: The range "20 to 40% of all maternal deaths" is quite wide. If this comes from different sources, clarifying why such a broad range exists would strengthen credibility.

Response: *We have corrected this in the revised manuscript.*

Results

Comment 9. Minor grammatical slips: "…leading to incorrect advice about breastfeeding. This prompted our desire to conduct this qualitative study…" → "prompted our decision" or "prompted us to conduct" would be smoother.

“…In contrast to medical or individual, the social model…” → should read “In contrast to the medical or individual model…”.

Response: *We have corrected this in the revised manuscript.*

Comment 10. Redundancy: Disability prevalence (4.3%) is mentioned in both the abstract and background. While not incorrect, it could feel repetitive unless tied to a distinct point in each section.

Response: *We have corrected this in the revised manuscript (removed from abstract).*

Comment 11. Framing of justification: The justification is present, but the link between the pilot trial results (48% reduction in mortality) and the qualitative study could be made sharper. Right now, the transition feels slightly abrupt—moving from trial outcomes to disability issues without a clear bridge.

Response: *We have corrected this section to clarify this.*

Methods

Comment 12. All my comments on this section have been well addressed. It is now logically structured, with clear subsections for setting, recruitment, data collection, and analysis. The restructuring improves readability and methodological transparency.

Response: *Thank you.*

Findings

Comment 13. Table 2. Theme summary should precede the presentation of the detailed thematic findings with a short narrative of what themes were identified before going into details of each theme with their quotes.

Response: *Thank you. We have moved Table 2 to the beginning of the Results section and added a short narrative summarizing the themes before presenting the detailed findings.*

Comment 14. Typo in the first paragraph of "Social stigmatisation of girls with disabilities" in "disability" spelling

Response: *We added corrected this in the revised manuscript.*

Comment 15. Overall, the section remains descriptive, but now sits within a clear framework, and the analytic unpacking is deferred to the Discussion.

Response: *Thank you.*

Discussion

Comment 16. Some subsections (Strengths and limitations and Conclusion and recommendation) have not been clearly demarcated. They seem to join the starting sentences. Format these appropriately.

Response: *We added corrected this in the revised manuscript.*

Comment 17. My comment has been fully addressed. The revised Discussion now has clear analytical inferences, policy/practice implications, regional comparisons, and a strengths/limitations subsection. The section is much stronger and aligns with qualitative reporting standards.

Response: *Thank you,*

Discussion

Comment 18. The comment has been fully addressed. The conclusion now goes beyond general advocacy and provides concrete, actionable recommendations for the Ministry of Health and other stakeholders.

Response: *Thank you,*
